# In Memoriam: Professor Nathaniel J. Szewczyk (January 28, 1973 – July 26, 2025)

**DOI:** 10.1038/s41526-026-00605-0

**Published:** 2026-06-02

**Authors:** Atsushi Higashitani, Timothy Etheridge

**Affiliations:** 1 Graduate School of Life Sciences, Tohoku University, Sendai, Japan; 2 Research Center for Space Cross-Tech, Tohoku University, Sendai, Japan; 3 Department of Physical Therapy, Ohio University, Athens, OH USA

**Keywords:** Climate sciences, Physics

## A global visionary in muscle physiology and space biology

The international space life sciences community mourns the passing of Professor Nathaniel J. Szewczyk, an eminent scholar whose work bridged the gap between fundamental molecular biology and the practical challenges of long-duration human spaceflight. Nate was a driving force in establishing the nematode *Caenorhabditis elegans* as a high-fidelity model for understanding human disease and physiological adaptation to microgravity.

Nate’s academic leadership spanned world-class institutions. He held the Osteopathic Heritage Foundation Ralph S. Licklider, D.O., Endowed Professorship in Molecular Medicine at Ohio University (Ohio Musculoskeletal and Neurological Institute, OMNI), following a distinguished tenure as Professor of Space Biology at the University of Nottingham. His foundational work was deeply rooted in his time at NASA Ames Research Center, where he played a pivotal role in one of the most extraordinary chapters of space biology.

Following the tragic atmospheric breakup of the Space Shuttle Columbia (STS-107) in 2003, Nate, alongside Dr. Catharine A. Conley and their colleagues, identified and recovered live *C. elegans* from the wreckage^1^. This profound discovery, documented in their seminal paper, *“Caenorhabditis elegans survives atmospheric breakup of STS-107, space shuttle Columbia”*, proved the resilience of life and the viability of the nematode as a robust model for space research.

Building on this legacy, Nate and Dr. Michel Viso (CNES) organized the International *Caenorhabditis elegans* Spaceflight Experiment 1 (ICE-First) in 2004^2^.^-^ This mission marked the beginning of a lifelong partnership between Nate and myself (A.H.)^3,4^. He provided dedicated support to researchers from many countries—including myself, from Japan, as well as from Canada, France, and beyond—by assisting with the preparation of *C. elegans* ICE-first samples in Toulouse, France, transporting and retrieving the samples to and from the Soyuz spacecraft, and the subsequent analyses.

**Fig. 1:**
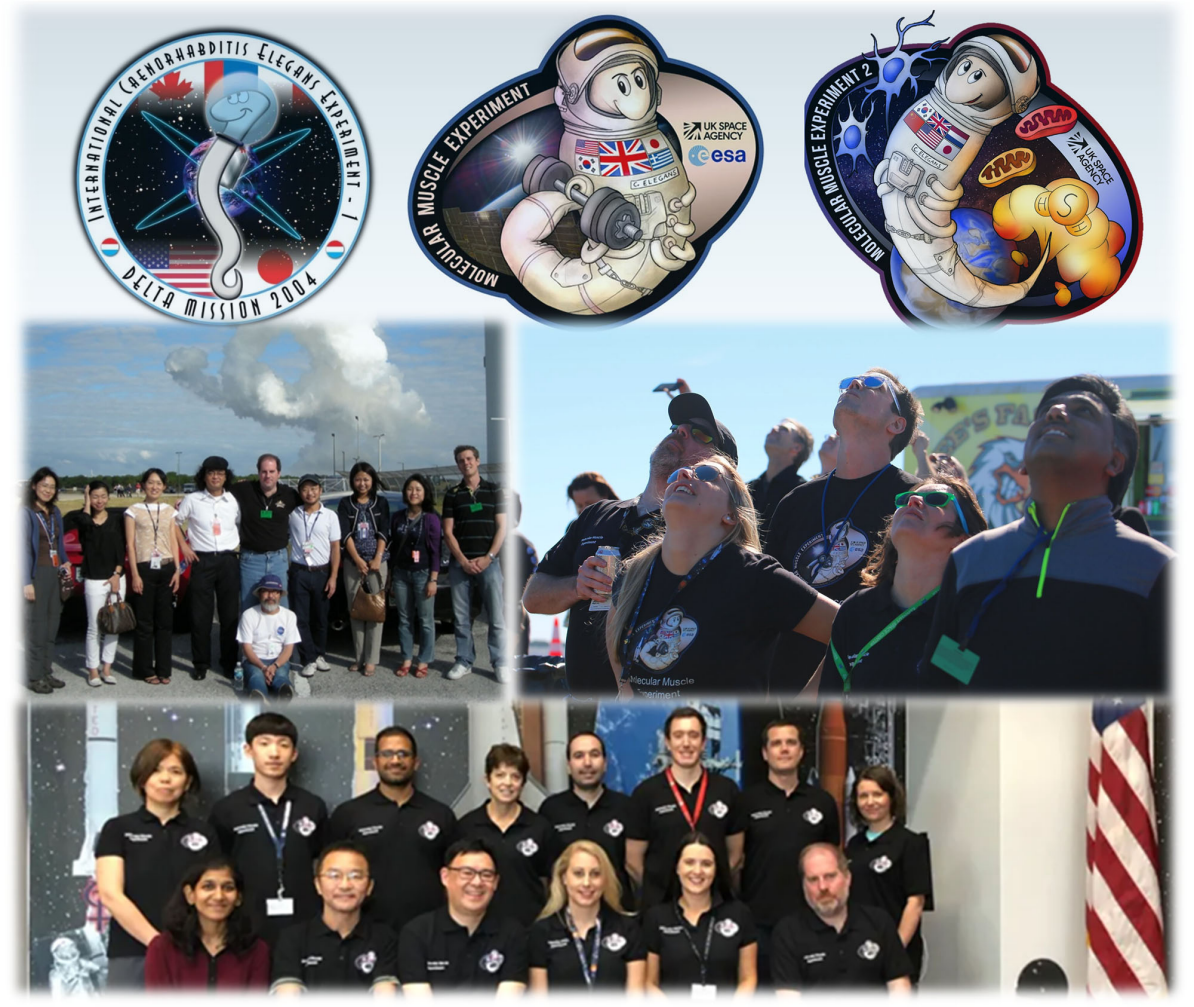
Selected photos of the international collaborative research team organized by Nate at Kennedy Space Center, along with decals for ICE-first and MME1 and 2.

Nate’s mentorship also fostered the careers of many, including that of Tim Etheridge – his first postdoctoral researcher^5,6^. In Molecular Muscle Experiments 1 and 2, Nate and I (T.E.) acted as Co-PI’s. Centered around the UK Space Agency, we led space experiments aiming to understand and prevent health decline during spaceflight using nematodes, in collaboration with numerous researchers from the UK, US, Greece, South Korea, Japan, and China^7–13^. Together, this international team spent decades advancing our understanding of age-related muscle atrophy and muscular dystrophy through the lens of spaceflight (Fig. 1)^14–19^.

Nate’s commitment to the next generation was unparalleled; he supported experiments ranging from high school projects to advanced doctoral studies with endless kindness and brilliance. As we look toward the future of lunar and Martian exploration, we do so on the foundation Nate built—a foundation of scientific rigor, international bridge-building, and an enduring curiosity about life’s resilience.

## Data Availability

No datasets were generated or analyzed during the current study.
